# Antitumour and biological effects of letrozole and GnRH analogue as primary therapy in premenopausal women with ER and PgR positive locally advanced operable breast cancer

**DOI:** 10.1038/sj.bjc.6603947

**Published:** 2007-08-21

**Authors:** R Torrisi, V Bagnardi, G Pruneri, R Ghisini, L Bottiglieri, E Magni, P Veronesi, C D'Alessandro, A Luini, S Dellapasqua, G Viale, A Goldhirsch, M Colleoni

**Affiliations:** 1 Research Unit of Medical Senology, European Institute of Oncology Milan, via Ripamonti 435, Milan 20141, Italy; 2 Division of Epidemiology and Biostatistics, European Institute of Oncology Milan, via Ripamonti 435, Milan 20141, Italy; 3 Department of Statistics, University of Milan Bicocca, Piazza dell'Ateneo Nuovo 1, Milan 20126, Italy; 4 Division of Pathology, European Institute of Oncology Milan, via Ripamonti 435, Milan 20141, Italy; 5 School of Medicine, University of Milan, via Festa del Perdono 7, Milan 20135, Italy; 6 Department of Medicine, European Institute of Oncology Milan, via Ripamonti 435, Milan 20141, Italy; 7 Medical Care Unit, European Institute of Oncology Milan, via Ripamonti 435, Milan 20141, Italy; 8 Division of Senology, European Institute of Oncology Milan, via Ripamonti 435, Milan 20141, Italy

**Keywords:** preoperative, primary therapy, premenopausal, letrozole

## Abstract

Preoperative endocrine therapy is effective in postmenopausal patients with breast cancers expressing oestrogen receptor. We investigated the activity of primary therapy with letrozole in combination with GnRH analogue in premenopausal women with T2–T4 N0–N2 breast cancer, whose tumours expressed oestrogen and progesterone receptors. We measured the expression of molecular factors involved in responsiveness to endocrine agents including ER*α*, EGFR, HER2, MAP kinases (and phosphorylated forms) ER-*β*1, both at initial biopsy and at the time of surgery. Thirty-five patients were included and 32 patients were evaluable for response. Sixteen patients (50%, 95% CI 32–68%) obtained a partial response, 16 patients were stable. One patient showed pathological complete response (3%, 95% CI 0–16%). Response was significantly associated with younger age (*P*<0.05) and a longer duration of treatment (*P*<0.05). Treatment significantly decreased ER*α*-p-Ser^118^ and upregulated ER-*β*1, independently of response. No or negligible overexpression of EGFR was observed at baseline or after treatment in this population. Preoperative letrozole and GnRH analogue are effective in premenopausal women. A biological response in terms of downregulation of phosphorylated ER*α* was observed in all patients. Future investigations might focus on treatments of longer duration.

Preoperative cytotoxic therapy has proved less effective in women with breast cancers expressing oestrogen receptor (ER) (Colleoni *et al*, 2004; Kaufmann *et al*, 2006). Endocrine therapy is a logical alternative. In postmenopausal women, neoadjuvant treatment with aromatase inhibitors showed promising results, better than those observed with tamoxifen (Eiermann *et al*, 2001; Miller *et al*, 2001; Smith *et al*, 2005; Abrial *et al*, 2006).

In premenopausal women, the use of aromatase inhibitors leads to an increase in gonadotropin secretion because of the reduced feedback of oestrogens on hypothalamus and pituitary and a subsequent stimulation of ovarian activity (Smith and Dowsett, 2003). The combination of aromatase inhibitors with GnRH analogue can obtain a complete oestrogen blockade by suppression of ovarian function and of peripheral oestrogen synthesis (Smith and Dowsett, 2003). However, limited experience has been described with aromatase inhibitors in combination with ovarian function suppression in premenopausal women with advanced breast cancer (Forward *et al*, 2004). Aromatase inhibitors in combination with GnRH analogues have not been systematically studied as preoperative therapy in premenopausal women with locally advanced operable breast cancer.

Mechanisms underlying responsiveness and/or resistance to aromatase inhibitors are poorly understood. It has been proposed that tumour cells may escape the inhibitory effects of antioestrogens by increasing ligand-independent phosphorylation of ER, possibly as a result of an overexpression of growth factors, namely the EGFR, Her-2/neu (HER2) and the IGF-IR pathways through increased levels of mitogen-activated protein kinases (MAPKs) (Chen *et al*, 2002; Lannigan, 2003). The low levels of the isoform *β* of ER have been associated with resistance to tamoxifen (Esslimani-Sahla *et al*, 2004; Hopp *et al*, 2004), but further data failed to show any association with response to preoperative toremifene (Cappelletti *et al*, 2004).

In the present study, we investigated the antitumour activity and expression of biological factors in a selected population of premenopausal patients with locally advanced breast cancer whose tumours expressed both ER and progesterone receptor (PgR) in at least 10% of tumour cells. Given the limited knowledge on the endocrine effects of aromatase inhibitors in premenopausal women, we also measured sequential 17-*β*-oestradiol levels in a subgroup of patients for whom repeated measurements were available.

## PATIENTS AND METHODS

Premenopausal patients with histologically proven T2–T4b breast cancer whose tumours showed ER and PgR immunoreactivity in ⩾10% of cells, consecutively admitted at the Department of Medicine of the European Institute of Oncology from January 2002 to April 2004 were enrolled in the study. All patients were submitted to a core biopsy using an 18 gauge needle of the primary tumour for histological diagnosis and assessment of biological characteristics (hormone receptor status, Ki-67 labelling index and immunoreactivity for HER2).

All patients were premenopausal as assessed by measurement of circulating oestradiol and gonadotrophins within the premenopausal range according to the reference value for each laboratory, and/or regular menses in the past 6 months. Investigations (chest X-ray, abdomen ultrasound and bone scan) were performed to exclude distant metastasis and blood tests were performed to assess bone marrow, renal and hepatic function within 2 weeks of inclusion in the study.

The primary tumour was measured clinically by calliper in two principal diameters and by breast ultrasound and mammography. Tumour response was assessed clinically every month and by breast ultrasound and mammography after 2 and 4 months of treatment. Clinical response was defined according to WHO criteria, as ⩾50% reduction of the product of two diameters of the tumour measured by calliper by the same examiner and by at least one medical imaging examination. Patients were submitted routinely to surgery after the second evaluation. A pathological complete remission (pCR) was defined as a total disappearance of the invasive tumour in both breast and axillary lymph nodes (Kuerer *et al*, 1999).

Endocrine therapy commenced with ovarian suppression obtained by means of a GnRH analogue, which was administered as intramuscular triptorelin 11.25 mg every 3 months. The aromatase inhibitor letrozole 2.5 mg per day was added when circulating oestradiol was in the postmenopausal range according to each laboratory reference value and was administered for at least 3 months. Oestradiol and gonadotrophin levels were repeated monthly during treatment with letrozole in order to verify the maintenance of ovarian function suppression.

Written informed consent was obtained from all patients. The Protocol was notified to the Ethical Committee.

This is a single institution study. All included patients had pathological evaluation performed at the EIO. Surgical specimens were extensively sampled for the evaluation of residual tumour as previously described (Colleoni *et al*, 2004). Immunostaining experiments for the localisation of ER (clone ID5, DakoCytomation, Glostrup, Denmark, pretreatment with EDTA, at 1 : 100 dilution) and PgR (636, DakoCytomation, EDTA, 1 : 400), Her2/neu protein (polyclonal antibody, DakoCytomation, EDTA, 1 : 800) and Ki-67 antigen (MIB-1, DakoCytomation, EDTA, 1 : 200) were performed on consecutive tissue sections of the diagnostic biopsies and residual tumour after surgery, if any, as previously reported (Colleoni *et al*, 2004).

Furthermore, we also investigated the prevalence and the predictive role of the immunoreactivity for phospho-ER*α* (Ser^118^) (pER*α*, polyclonal antibody, Cell Signaling Technology Inc. (Danvers, MA, USA) EDTA, 1 : 100), ER-*β*1 (PP-G5/10, DakoCytomation, citrate, 1 : 20), epidermal growth factor receptor (EGFR, 31G7, Zymed Laboratories (San Francisco, CA, USA) pronase, 1 : 20), phospho-EGFR (p-EGFR, polyclonal antibody, Cell Signaling, EDTA, 1 : 400), phospho-HER2 (p-HER2, polyclonal antibody, DakoCytomation, EDTA, 1 : 200), phospho-p38 (p-p38MAPK, 12F8, Cell Signaling, citrate, 1 : 100) and phospho–p44/42 (p-p44/42MAPK, 20G11, Cell Signaling, citrate, 1 : 100) MAP-kinases in a subgroup of 27 patients for whom tumour tissue from both diagnostic biopsies and post-treatment surgical specimens was available.

In each case at least 200 neoplastic cells were evaluated at × 400 magnification and the percentage of cells showing any definite nuclear (for ER, PgR and Ki-67) and membranous (for EGFR, HER2 and their phosphorylated forms) immunoreactivity was recorded.

The immunostained slides were evaluated independently by two of the authors. The thresholds for hormone receptors (ER and PgR) and Ki-67 labelling index were 10 and 20%, respectively, according to published studies and the routine practice. The value of 20% for proliferative activity was selected based on previous data from our group indicating that this threshold significantly correlated with higher response rate to preoperative chemotherapy (Colleoni *et al*, 2004). For HER2, we used the FDA approved score system.

Finally, cutoffs for p-ER*α*-Ser^118^, ER-*β*, EGFR, pEGFR, pHER2, p-p-38 (MAPK) and p-p44/42 (MAPK) have not been defined yet, since they have not been extensively investigated and are not used in the routine clinical practice, therefore samples containing any percentage of stained cells were considered positive.

The main end point of this study was clinical response. According to literature (Eiermann *et al*, 2001; Gazet *et al*, 2001; Smith *et al*, 2005), we expected to observe at least 45% of clinical responses. We adopted an optimal two-stage design, with a target rate of clinical responses of 45% deemed acceptable, and a response rate of 22% considered unacceptable.

For a 0.1 type I error and a 0.1 type II error probabilities, 17 patients were needed in the first stage and if 4 or less achieved an objective response the study had to be closed for insufficient activity. If 5 or more objective responses were observed among these initial patients, an additional 13 assessable patients had to be entered. If 10 or more objective responses were observed among the 30 assessable patients, the treatment would be considered worthy of further consideration.

The Mann–Whitney *U*-test and Fisher's exact test were used to evaluate the differences, respectively, in continuous and categorical variables between responders and non-responders.

The one-sample Wilcoxon signed-rank test and McNemar's Test were used to examine the change of expression, respectively, in continuous and categorical molecular parameters, among all tumours, between the pretreatment biopsy and surgery.

The change of expression was also evaluated in responders and non-responders, and differences between groups were also tested using the Mann–Whitney *U*-statistics.

To assess whether oestradiol levels decreases or increases during treatment, a linear mixed models for repeated-measures data were used. In the regression analysis, oestradiol levels were logarithmically transformed to reduce skewedness.

Disease-free survival (DFS) was calculated from the date of surgery to any relapse, the appearance of a second primary cancer, or death, whichever occurred first.

Estimation of the DFS was performed using the Kaplan–Meier method, and the log-rank test was used to assess the survival difference between responders and non-responders.

All analyses were performed with the SAS software (SAS Institute, Cary, NC, USA). All tests were two sided.

## RESULTS

Thirty-nine consecutive patients were considered for the study and 37 were eligible. Two patients were excluded, one because of synchronous bone metastasis and one because of multifocal tumour with different receptor status.

Among eligible patients five patients were considered not evaluable for response (two refused further treatment, one received anastrozole instead of letrozole and two did not achieve postmenopausal status within 3 months and were submitted to surgery).

Thirty-two patients were therefore considered evaluable for response. Median age was 41.5 years (range, 33–54 years). Twenty-eight patients received 3-month triptorelin and four patients received 28-day goserelin. Median duration of total treatment from initiation of GnRH analogue was 5.2 months (IQ range, 4.6–5.6), while median duration of letrozole was 4 months (IQ range, 3.4–4.4).

Table 1 reports the clinical and biological characteristics of the tumours at the pretreatment biopsy and at surgery. All but one tumour maintained ER positivity at surgery, while downregulation of PgR was observed in 26 patients. Treatment also induced downregulation of proliferative activity with 9 (28%) patients showing a Ki-67 >20% at surgery as compared with 17 (53%) patients with high proliferative activity in initial biopsy (McNemar's test *P*<0.05). No change was observed in HER2 overexpression.

**Figure 1:**
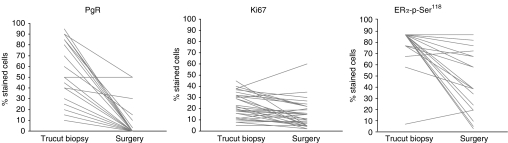
Change between pretreatment and post-treatment levels of PgR, Ki-67 and for ER*α*-p-Ser^118^ in each patient.

The changes between pretreatment and the post-treatment values for PgR and Ki-67 in each patient are reported in Figure 1.

One patient (3%) obtained a complete clinical response, which was confirmed as a pCR at pathological examination. Fifteen patients (47%) obtained a clinical and imaging partial response giving an overall response rate of 50% (95% CI 32–68%). Sixteen patients were stable and no patient progressed during treatment.

Breast-conserving surgery was performed in 15 patients (47%) while 17 patients (53%) were submitted to mastectomy. Twenty-two patients received anthracycline-containing chemotherapy as adjuvant treatment, while 10 patients continued endocrine therapy.

Median follow-up was 36 months (range, 6–51). We observed seven recurrences (one locoregional and six distant recurrences) and two deaths. Three-year DFS was 76% (95% CI 59–93%) (83% in clinical responders, 70% in non-responders, log-rank test *P*-value 0.23.).

Univariate analysis showed that clinical response was positively associated with duration of letrozole treatment which lasted 4.2 months (IQ range, 3.7–4.7) for clinical responders *vs* 3.4 months (IQ range, 2.0–5.4) for patients obtaining stable disease (Mann–Whitney *U*-test *P*<0.05). Clinical response was positively associated also with age, since median age in responding patients was 39 *vs* 44.5 years for non-responding patients (Mann–Whitney *U*-test *P*<0.05). Twelve (67%) patients with PgR levels ⩾70%, corresponding to the median value of PgR, and four patients (29%) with PgR <70% had a clinical response (Fisher's exact test *P*=0.07).

**Figure 2:**
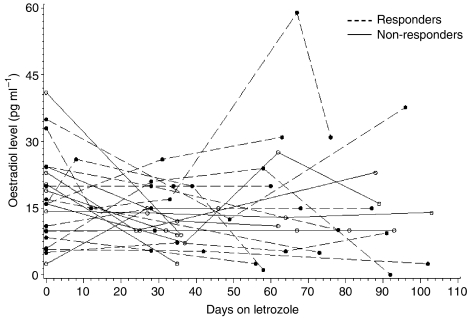
Time course of oestradiol levels upon treatment with letrozole according to clinical response. Time 0 is represented by the oestradiol levels obtained after GnRH analogue. Dashed line represents oestradiol time course in clinical responders and continuous line in non-responders.

Twenty-three patients had repeated measurements of oestradiol at different time points during treatment. After GnRH analogue median oestradiol was 16 pg ml^−1^ (IQ range, 10–23). Linear mixed regression for repeated-measures data showed that there was no significant change in oestradiol levels upon treatment with letrozole (Figure 2). The oestradiol time course was not correlated with clinical response (Figure 2). Gonadotrophins were measured at the same time points of oestradiol and confirmed the suppression of LH and FSH.

Treatment was well tolerated and no patient discontinued therapy because of side effects. Main toxicities are reported in Table 2.

Molecular analysis was performed in 27 patients for whom tumour tissue from either pretreatment biopsy or definitive surgery was available. High levels of ER*α*-p-Ser^118^ were detected in all patients and were significantly downregulated after treatment (Wilcoxon signed-rank test *P*<0.0001; Table 2). The change between pretreatment and the post-treatment values in each patient for ER*α*-p-Ser^118^ are reported in Figure 1.

Oestrogen receptor-*β*1 (ER-*β*1) immunoreactivity was detectable in 24 patients and was significantly upregulated after treatment (Wilcoxon signed-rank test *P*<0.05; Table 3).

Neither EGFR nor its phosphorylated form (p-EGFR) was detectable in any sample in the pretreatment biopsy, while after treatment very few cells (median value 5% range, 3–10%) positively stained for EGFR in five surgical specimens. Phosphorylated HER2 was expressed in 10 patients (37%) at baseline. Four out of five patients with HER2 IHC 3 + stained positively for p-HER2 Phosphorylated-p38 (MAPK) was detected in 11 patients and p-p44/42 (MAPK) was detected in all patients except 2 at baseline and their levels remained substantially unchanged after treatment (Table 3).

Univariate analysis failed to show any association between baseline values of any molecular parameter and clinical response. No significant difference between baseline and post-treatment values were observed for any parameter among responding and not responding patients (Table 3).

## DISCUSSION

Preoperative treatment of patients with endocrine-responsive breast cancer represents a challenge for patients with the disease, medical oncologists, surgeons and pathologists. Despite the addition of taxanes to anthracyclines, the pCR rate after preoperative chemotherapy in hormone receptor-positive tumours remains relatively low (Kaufmann *et al*, 2006).

Primary endocrine therapy is able to induce a high rate of objective remissions and breast-conserving surgery (Abrial *et al*, 2006). Preoperative endocrine therapy has been historically restricted to postmenopausal women. Data arising from very small studies have been reported in premenopausal women treated with preoperative GnRH analogue (Gazet *et al*, 2001; Miller *et al*, 2001). Aromatase inhibitors in combination with GnRH analogue have not been systematically studied as primary therapy in premenopausal women with hormone receptor positive locally advanced operable breast cancer. Experience in advanced disease is also limited, ranging from anecdotal reports to small phase II studies (Forward *et al*, 2004; Dowsett *et al*, 2005). The present study showed that endocrine therapy inducing a maximal oestrogen blockade is feasible in premenopausal women and effective in terms of response rate (50%, 95% CI 32–68%). Results are in line with those already reported in postmenopausal women with aromatase inhibitors where the response rate ranged from 24 to 35%, when considering ultrasound evaluation (Eiermann *et al*, 2001; Smith *et al*, 2005). In our study, one patient (3%) achieved a pCR and 47% of patients were submitted to breast-conserving surgery. These figures too are comparable with those reported in randomised trials with preoperative aromatase inhibitors in postmenopausal patients. Preoperative endocrine therapy has been associated with a very low rate of pCR ranging from 1 to 8% (Kaufmann *et al*, 2006). The value of pCR as surrogate end point of clinical outcome in ER-positive tumours after primary chemotherapy, which was called into question in former studies, was recently confirmed by the results of a large retrospective study showing that pCR was positively associated with outcome in both ER-negative and ER-positive tumours (Ring *et al*, 2004; Guarneri *et al*, 2006), but no data after primary endocrine therapy are available. A significantly increased response rate was reported with 6 months as compared with 3 months of preoperative letrozole therapy (Paepke *et al*, 2006). It may therefore be that a higher pCR rate might be observed with prolonged treatment in the preoperative setting.

As expected, treatment induced a significant downregulation of both PgR expression and proliferative activity, although these were not significantly correlated with clinical response (Miller *et al*, 2006), although a trend for a greater reduction of Ki-67 was observed in responders.

Our results confirm a possible role for the expression of PgR in determining the endocrine responsiveness of the tumour. High levels of PgR were positively associated with the occurrence of a clinical response if compared with lower levels although the difference was of borderline significance.

An important issue relates to the extent of oestrogen suppression occurring upon treatment with an aromatase inhibitor in combination with a GnRH analogue in premenopausal women. Available data range from anecdotal reports to small phase II studies and include mainly the second-generation steroidal inhibitor formestane other than the non-steroidal inhibitors anastrozole and vorozole. In all reports, a further inhibition of oestradiol levels was observed after therapy with aromatase inhibitor (Dowsett *et al*, 1992, 1999; Celio *et al*, 1999; Forward *et al*, 2004). In our study, we evaluated the levels of oestradiol at different time points during the study to determine the hormonal status. We acknowledge that our data are limited because of the heterogeneity of time points of the determinations and the multiple laboratories employed, other than the lack of adequately sensitive test for measuring oestradiol. We found that oestradiol levels were maintained in the postmenopausal range. We observed a slight and not significant further suppression of oestradiol after letrozole administration only in 13 patients out of 23. Whether the extent of oestradiol suppression may affect the clinical response is currently unknown. Although our data in a limited number of patients failed to show any correlation between oestradiol time course and clinical response, the issue of oestrogen suppression in premenopausal women receiving aromatase inhibitors needs to be further addressed, given these drugs are currently investigated in the adjuvant setting in large phase III trials. On the other hand, two patients failed to reach a full-ovarian function suppression within 3 months after the first administration of 3-month triptorelin as documented by persisting menstrual cycle and oestradiol levels within the range of premenopause and were submitted to surgery. The failure of the 3-month GnRH analogue formulation in achieving ovarian function suppression has been previously reported either in breast and in prostate cancer patients (Yri *et al*, 2006; Schmid *et al*, 2007).

Few data are available on the molecular changes occurring *in vivo* after neoadjuvant therapy with aromatase inhibitors and their predictive value on clinical response. Our study population was highly selected according to the expression of both steroid hormone receptors. Molecular analysis showing high baseline levels of ER*α*-p-Ser^118^ and the absent or negligible expression of EGFR and HER2 in all patients emphasise the endocrine-sensitive nature of our population.

Almost all available data on mechanisms of resistance to aromatase inhibitors are derived from preclinical models using long-term oestrogen-deprived (LTED) cells which supposedly mimic the oestrogen depletion seen clinically with letrozole (Brodie *et al*, 2005; Dowsett *et al*, 2005). In these models, an upregulation of the ER*α*-p-Ser^118^ following an activation of growth factor pathway signalling (EGFR, IGFR) and the activation of MAP kinase has been demonstrated when tumours started to grow despite letrozole (Chen *et al*, 2006).

Ser^118^ represents the major site of phosphorylation of ER upon binding with oestradiol (Lannigan, 2003). Baseline levels of ER*α*-p-Ser^118^ have been positively associated with favourable tumour characteristics, with a better outcome after therapy with tamoxifen and with expression of PgR (Murphy *et al*, 2004a, 2004b). It has been hypothesised that, as distinct from what occurs *in vitro*, ER*α*-p-Ser^118^ is a marker of a functional ligand-dependent pathway in primary breast tumours and is involved in the mechanisms by which the oestradiol/ER*α* complex regulates downstream signalling (Murphy *et al*, 2004b).

We may speculate that the downregulation of the ER*α*-p-Ser^118^ we observed after treatment is a consequence of the suppressed levels of circulating oestrogens, suggesting the occurrence of a reduced oestrogen-induced transcription, leading finally to a clinical response. In a previous study with gefitinib plus or minus anastrozole, the decrease of ER*α*-p-Ser^118^ was associated with clinical response (Polychronis *et al*, 2005). Therefore, we might have expected that non-responders should not experience a decrease of ER*α*-p-Ser^118^, but rather an increased phosphorylation as a consequence of a ligand-independent activation which is a putative mechanism of endocrine resistance (Brodie *et al*, 2005; Chen *et al*, 2006). However, in our study no difference in the extent of the decreased phosphorylation was observed in responders or non-responders, and we saw no increase in EGFR or HER2 expression or in phosphorylated MAP kinase activity in the clinical non-responders. It is possible that our non-responders were not inherently resistant, but merely not treated for long enough.

Our results on the correlation between biomarkers and clinical response, however, should be considered with caution given the small number of patients included in this analysis.

The significance and the role of ER-*β* and the interaction with ER-*α* are unclear. The ratio ER*α* : ER-*β* within tumour cells has been hypothesised to play a functional role in cell growth (Shaw *et al*, 2006). Conflicting data have been reported on the role of ER-*β* either *in vitro* and *in vivo*, in that results from preclinical and clinical studies have associated ER-*β* either positively (protein expression) or negatively (mRNA) with prognosis and response to endocrine treatment (Cappelletti *et al*, 2004; Saji *et al*, 2005). The results of our study, showing a significant upregulation of ER-*β* after treatment irrespective of clinical response, do not help to get further insight into this issue.

In conclusion, the results of the present study indicate that therapy with aromatase inhibitors, in combination with a GnRH analogue, is safe and effective in premenopausal women with locally advanced operable breast cancer. A biological response, in terms of downregulation of the oestrogenic signalling, was observed in all patients, and clinical response in 50% of the patients. The trend to improved response rate observed with longer duration in the present study and in previously published studies support the development of tailored endocrine therapy of longer duration in selected populations of premenopausal patients with endocrine-responsive tumours.

## Figures and Tables

**Table 1:** Clinical and biological characteristics of tumours at baseline and at surgery

	**Trucut biopsy**	**Surgery**
Evaluable patients	32	32^a^
*Clinical stage*		
T2/T3	24/5	—
T4b	3	
N0/N1	7/25	
		
*Pathological stage*		
T0	—	1
T1c/T2		10/16
T3/T4		4/1
N0/N1		3/6
N2/N3		12/5
		
*ER status*		
Negative	0	1
10–49%	3	4
⩾50%	29	26
		
*PgR status* b		
Negative	0	25
10–49%	11	3
⩾50%	21	2
		
*Ki-67*		
⩾20%	17	9
<20%	15	22
		
*HER2 overexpression*		
3+	5	5
2+	4	6
1+/absent	23	20
EGFR expression^c^	0	5

a One patient was not evaluable for biological characteristics at surgery because she achieved a pCR.

b One patient with PgR <10% was included among PgR negative.

c Tumours expressing any percentage of stained cells were considered positive.

**Table 2:** Main toxicities

**Toxicity (*N*=30)**	**G1 *N* (%)**	**G2 *N* (%)**
Hot flashes	**3** (10%)	**17** (56.7%)
Arthromyalgia	**5** (16.7%)	**2** (6.7%)
Headache	**4** (13%)	0
Night sweating	**2** (6.7%)	**2** (6.7%)
Rash	**1** (3.3%)	**1** (3.3%)
Nausea	**1** (3.3%)	**1** (3.3%)
Neurological	**2** (6.7%)	0
Astenia	**1** (3.3%)	0
Dizziness	**1** (3.3%)	0

**Table 3:** Basal levels and changes of molecular parameters after treatment according to clinical response on 27 evaluable patients

	**No. of positive samples at trucut**	**Median value at trucut^a^ [IQR]**	**No. of positive samples at surgery**	**Median difference between trucut and surgery [IQR]**	***P*-value**
*ER*α-*p*-*Ser*^*118*^					
Total	27/27	90 [90, 90]	26/26	−25 [−65, −5]	<0.001^b^
CR	13/13	90 [90, 90]	12/12	−25 [−55, −5]	0.68^c^
NR	14/14	90 [90, 90]	14/14	−25 [−75, −5]	
					
*pHER2*					
Total	10/27	0 [0, 5]	8/26	0 [−5, 0]	0.49^b^
CR	6/13	0 [0, 5]	5/12	0 [0, 0]	0.73^c^
NR	4/14	0 [0, 3]	3/14	0 [−5, 0]	
					
*ER-β*					
Total	23/27	48 [5, 90]	23/26	10 [0, 60]	0.02^b^
CR	11/13	60 [5, 90]	10/12	10 [0, 60]	0.66^c^
NR	12/14	41 [5, 80]	13/14	9 [−10, 42]	
					
*p*-*p38*					
Total	11/27	0 [0, 3]	13/26	0 [−3, 20]	0.10^b^
CR	5/13	0 [0, 3]	5/12	0 [−3, 20]	0.68^c^
NR	6/14	0 [0, 3]	8/14	0 [−3, 12]	
					
*p*-*p44*/*42*					
Total	23/27	10 [5, 15]	18/26	−5 [−10, 28]	0.78^b^
CR	12/13	10 [10–15]	8/12	−3 [−10, 30]	0.66^c^
NR	11/14	9 [3–10]	10/14	−5 [−10, 24]	

CR=clinical responders; IQR=interquartile range; NR=non-responders.

a Values are expressed as percentage of stained cells.

b Change of the expression of molecular parameters among all tumours assessed by the Wilcoxon signed-rank test.

c Change of the expression of the molecular parameters between clinical responders and non-responders assessed by the Mann–Whitney *U*-test.
